# Bacterial profile of wound site infections and evaluation of risk factors for sepsis among road traffic accident patients from Apex Trauma Centre, Northern India

**DOI:** 10.1099/acmi.0.000836.v4

**Published:** 2024-10-14

**Authors:** Aparna Singh, Sangram Singh Patel, Chinmoy Sahu, Amit Kumar Singh, Nidhi Tejan, Gerlin Varghese, Ashima Jamwal, Pooja Singh, Malay Ghar

**Affiliations:** 1 Department of Microbiology, Sanjay Gandhi Post Graduate Institute of Medical Sciences, Lucknow, Uttar Pradesh, India; 2 Speciality of Trauma Surgery, Apex Trauma Centre, Sanjay Gandhi Post Graduate Institute of Medical Sciences, Lucknow, Uttar Pradesh, India

**Keywords:** bacterial profile, risk factors, RTA wound, sepsis, wound infection

## Abstract

**Background.** Among the most significant yet often ignored health issues worldwide are trauma and accidental injuries. India accounts for 11% of global deaths in road accidents, the highest in the world, according to the World Bank report. There are limited data about the bacterial contamination of road traffic accident (RTA) wounds and their antibiotic susceptibility patterns.

**Materials and Methods.** This prospective study was conducted in a tertiary care centre in northern India from January 2023 to January 2024. Wound deep swabs or aspirates were collected from RTA patients with traumatic injuries at different time intervals. Gram stain and culture were performed, and positive aerobic culture was subjected to antibiotic susceptibility testing. Organism identification was done using MALDI-TOF MS and routine biochemical tests. Blood samples were also collected to rule out bloodstream infections during follow-up if the patient became febrile or showed symptoms of systemic infection. Sepsis was defined in those patients who had two or more scores in the systemic inflammatory response syndrome criteria with a positive microbiological culture. Risk factors were evaluated for sepsis on the basis of the patient’s vitals, injury characteristics, procalcitonin, Glasgow Coma Scale (GCS) score, need for mechanical ventilation and complete blood count, which were obtained from the patient’s admission file.

**Results.** A total of 189 wound samples were collected, of which 99 (52.38%) samples showed the growth of microorganisms. The aerobic isolates included 69 (69.69%) Gram-negative bacilli, of which the majority were *Klebsiella pneumoniae*, 28 (28.28%) Gram-positive cocci, of which the majority were *Staphylococcus aureus* and 2 (2.02%) anaerobic isolates. Among the Gram-negative isolates, none of the isolates were resistant to colistin. All *S. aureus* isolates were susceptible to vancomycin, teicoplanin and levonadifloxacin. Sepsis developed in 50 (26.45 %) patients. Significant risk factors evaluated for sepsis were a raised procalcitonin level, a low GCS score, a higher injury severity score, the need for mechanical ventilation and a raised quick sequential organ failure assessment score.

**Conclusion.** It is essential to ascertain the profile of microorganisms isolated from RTA wounds in order to reduce antibiotic resistance and deliver efficient treatment.

## Highlights

- The prevalence of bacterial infection among road traffic accident patients with wounds was 52.38%.
- Infection by multidrug-resistant isolates (62.88%) and carbapenem-resistant isolates (52.17%) was high.
- Risk factors evaluated for sepsis, which were found to be significant in this study, were a low Glasgow Coma Scale score, a raised procalcitonin level, a higher injury severity score, the need for mechanical ventilation and a raised quick sequential organ failure assessment score.

## Data Summary

The SPSS statistical software (IBM SPSS version 20, Armonk, NY) was used for statistical analysis. No other supporting or new data, tools, software or code has been generated for this manuscript.

## Introduction

Road traffic accident (RTA) is any injury caused by a crash that begins, ends or involves a vehicle either fully or partially on a public road, in which at least one person is injured or killed [1]. According to the World Health Organization, 1.19 million RTA deaths occur annually, which is a significant rate globally [2]. Twenty to fifty million individuals each year sustain non-fatal injuries, many of which result in disability [3]. One of the top three causes of death for people in the age range of 5–44 years is vehicular traffic injuries [4].

A wound is an injury to living tissue that results from a cut, blow or other impact; these wounds usually involve broken or cut skin. Patients who have experienced trauma and developed an infection have been found to have a fivefold increased mortality rate compared to those who do not [5]. Worldwide, over 80% of late deaths among adult trauma patients are caused by infections [6]. Trauma that compromises the integrity of the skin and tissues endangers the host’s natural defences [5]. Patients suffering from trauma are more susceptible to infection due to a weakened immune system, compromised skin barrier integrity and invasive treatments carried out while they are in the hospital [5].

In 2016, a new definition was proposed by the European Society of Intensive Care Medicine and the Society of Critical Care Medicine: Sepsis-3 is a life-threatening organ dysfunction caused by a dysregulated host response to infection [7]. A healthy immune system and the preservation of colonization and resistance against bacterial infections are essentially dependent on the integrity of the intestinal barrier and the intestinal microbiome [8]. Barrier dysfunction of the intestinal mucosa, bacterial translocation and subsequent infections into the bloodstream are favoured by trauma, perfusion injury or surgery [9]. Prolonged use of antibiotics can cause dysbiosis and the growth of multiresistant bacteria, which can result in serious infections and problems after stressful events [10].

Thus far, the primary approaches to preventing trauma-related infections and sepsis have been to prevent organ failure (via temporary intravascular shunts, medicines, surgical care, prophylactic antibiotics, tetanus vaccination, etc.) and infections [11]. The three areas of greatest therapeutic interest are early injury severity detection, post-traumatic complication risk factor prediction and secondary damage prevention. The best supplement for early detection of possible problems such as infection is serum indicators of inflammatory response [12,14]. Leucocytosis and the release of acute-phase proteins, such as cytokines and C-reactive protein, are useful indicators of systemic inflammatory responses [15,17]. It has been demonstrated that procalcitonin (PCT) is a sign of sepsis and bacterial infection [18,19], and as a result of bacterial infection, PCT is secreted systemically from several types of cells outside the thyroid [20].

Sepsis in trauma patients was associated with an increased overall length of hospital and intensive care unit (ICU) stay as well as higher rates of single or multiple organ failure (MOF) [21,22]. Several risk factors associated with the development of sepsis among trauma patients have been identified. These include injury severity score (ISS), lower Glasgow Coma Scale (GCS) score, pre-existing medical conditions, revised trauma score (RTS), age, male gender, number of red blood cell (RBC) units transfused and the number of operative procedures [21,24].

Quick sequential organ failure assessment (qSOFA) is a score that has been proposed by the Sepsis-3 task force as a means of identifying patients who are at risk of sepsis and who are not in ICUs. This is the third international consensus definition for sepsis and septic shock [7]. According to the findings of a recent cohort study, qSOFA was a more accurate predictor of in-hospital mortality for septic patients in emergency departments compared to systemic inflammatory response syndrome (SIRS) [25].

To the best of our knowledge, this is the first study from India that has evaluated both the bacterial profile of wound site infections as well as the risk factors for sepsis among RTA patients.

## Rationale for Investigation

This prospective study was carried out to determine the bacterial profile and antibiogram of wound isolates among RTA patients. Risk factors that contributed to the development of sepsis were also assessed, given the paucity of data in this area of the trauma centre. Treatment of individuals with multiple organ injuries is particularly difficult because of the complex immune response in addition to varying injury patterns and severity [26]. Post-traumatic hyper-immunomodulation is associated with a higher risk of death in trauma patients during the first few days in the ICU and frequently results in post-injury complications, such as MOF or sepsis [27]. Hospitalized septic trauma patients continue to have a significant mortality rate (19.5–23%). Early intervention to stop the onset of sepsis can help patients obtain additional therapy and have better results [28].

## Objectives

There is a great need for the trauma care specialist to know the incidence, risk factors and the spectrum of pathogenic microorganisms and their antibiotic susceptibility causing wound site infections among RTA patients. The main aim of this study was to study the bacterial profile and antibiotic susceptibility pattern of aerobic wound culture isolates.

The objectives of this study were as follows:

- To study the aerobic and anaerobic profiles of wound site bacterial isolates among the RTA in-patients.
- To study the changes in the pattern of bacterial aetiology, with follow-up of patient samples for up to 2 weeks of hospitalization or until discharge, whichever occurred earlier.
- To study the risk factors for patients who develop bloodstream infections (BSI) or sepsis.
- To detect the antibiotic susceptibility pattern of only aerobic wound site bacterial isolates.

## Methods

### Study design

A prospective observational study was conducted where patients with wounds following RTA were investigated, and the risk factors were evaluated in those patients who developed sepsis during the study period. Approval for the study protocol was obtained from the Institutional Ethics Committee (IEC code: 2023-109-MD-131).

### Setting

**Fig. 1.**
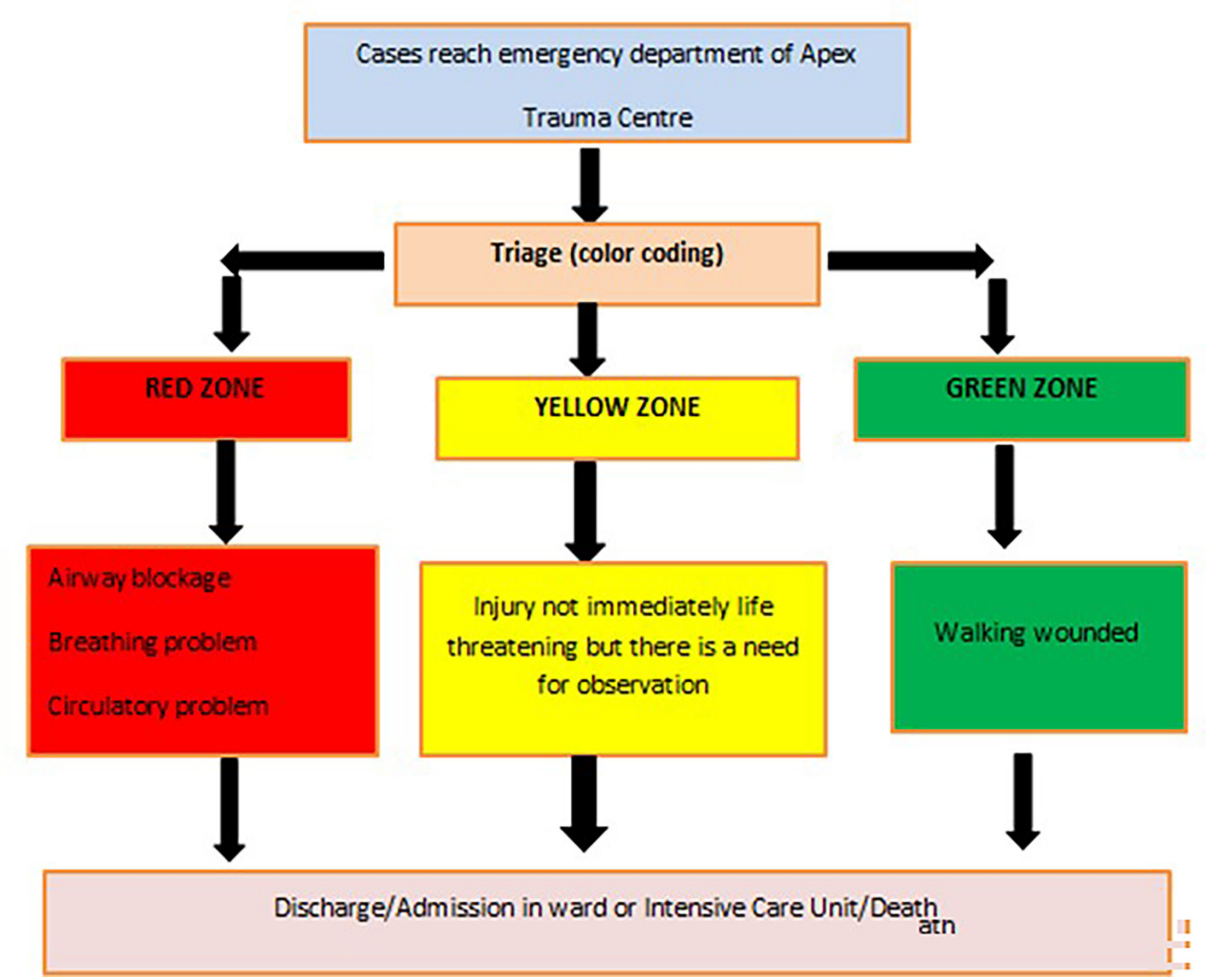
Flow chart showing the ATC triage care.

This study was carried out at the bacteriology section of the Department of Microbiology in collaboration with the Apex Trauma Centre (ATC) at a 1600-bedded tertiary care centre in northern India for the duration of January 2023 to January 2024. Informed written consent was obtained from all the patients of RTA attending the ATC and meeting the inclusion criteria of the study and was attended according to the triage care (Fig. 1). If the patient could not give consent due to a severe injury, informed consent was obtained from the legal guardian. Patients with airway and circulatory dysfunction were admitted to the red zone, patients requiring only observation but not admitted with life-threatening injuries were attended in the yellow zone and ambulatory patients were admitted to the green zone of the trauma centre. The type of wound, location of the wound, presence of wound contamination, antibiotic prophylaxis and vitals at the time of admission were noted. Wound deep swabs or aspirates were cultured, and their antibiotic susceptibility pattern was assessed. In those patients with BSIs, a blood culture was also performed. Wound infection was established in the patients when they had clinical characteristics such as fever, a raised total leucocyte count and local signs of inflammation at the wound site along with a positive bacterial culture of the wound isolate.

### Participants

The participants of the study included all patients of RTA with wounds ≥12 years of age except for the pregnant females.

#### Inclusion criteria

All RTA in-patients aged ≥12 years admitted to ATC within 24 h of trauma having traumatic wound injury were included in this study.

#### Exclusion criteria

Patients admitted to ATC after 24 h of trauma having traumatic wound injury, patients with trauma due to reasons other than RTA, those with <12 years of age, pregnant women and those not willing to give consent were excluded from this study.

### Variables

Wound deep swabs or aspirates were collected aseptically from RTA in-patients with sterile swabs tipped with dacron or rayon polyester, labelled properly and transported immediately to the laboratory without any delay. Wound samples were collected four times during hospitalization. The first sample was collected within 48 h of traumatic injury, the second sample was collected within 96 h, the third sample was collected in the first week and the fourth sample was collected in the second week. Patients were only followed up until 2 weeks of hospitalization or until discharge, whichever was earlier. Three wound deep swabs were collected each time in the case of the swab sample.

Blood samples were also collected to rule out BSIs during follow-up of hospitalized patients if the patient became febrile or showed symptoms of systemic infection. Two sets of blood cultures were collected for both the aerobic and anaerobic blood cultures.

#### Aerobic culture processing

One swab was used for smear preparation, and staining of the smear was done by Gram stain followed by microscopic examination of the stained smear at 100× magnification under an oil immersion lens. The second swab was used for aerobic bacterial culture by inoculation onto various culture media such as blood agar and MacConkey agar, and the inoculated plates were incubated aerobically for 24 h at 37 °C. The next day, growth was observed, and identification was performed using standard protocols, including Gram stain, motility test, biochemical tests such as catalase, oxidase, coagulase, indole, methyl red, Voges–Proskauer, citrate, urease and phenylalanine deaminase test and MALDI-TOF MS (Biomerieux). If no growth was found after 24 h, the culture plate was further incubated for another 24 h. No growth was declared only after incubating culture media for 48 h and was reported as sterile.

Antibiotic susceptibility testing was performed by the Kirby–Bauer disc diffusion method and interpreted according to M-100 edition-34 Clinical Laboratory Standards Institute (CLSI) guidelines except for the colistin and cefoperazone–sulbactam [29]. Broth microdilution was performed for colistin and interpreted according to the CLSI M-100 edition-34. The European Committee on Antimicrobial Susceptibility Testing version 14.0 guideline was followed for cefoperazone–sulbactam, in which the disc diffusion testing was performed and interpreted [30]. A multidrug-resistant organism (MDRO) was identified as having resistance to three or more classes of antibiotics.

#### Anaerobic culture processing

The third wound swab was used for anaerobic culture and processing. For anaerobic microorganism cultivation, the sample was kept in Robertson’s cooked meat broth and incubated, and growth was indicated by the turbidity of the broth. Turbid broth was then cultured on Wilkins–Chalgren anaerobic agar, and subsequently, a metronidazole antibiotic disc was placed over the streaked culture plate. An anoxomat anaerobic jar system was used for anaerobic bacterial cultivation, and then culture plates were incubated at 37 °C. After 48 h of incubation, the growth of the organism was observed, and organism identification was done using MALDI-TOF MS (Biomerieux). In cases of no growth, the culture plate was further incubated, and if no growth was observed for 5 days, then it was reported as sterile.

#### Wound infection criteria

Wound infection was established in patients when there was the presence of clinical signs and symptoms of infection such as fever, raised total leucocyte count, pain, redness, swelling, purulent exudate and foul smell, along with the culture-confirmed wound swab or aspirate sample.

#### Injury severity score

The Abbreviated Injury Scale grading system was used by Baker *et al*. to propose the ISS scale, which divides injury locations into six categories: head and neck, face, chest, abdomen and pelvis, limbs and pelvis and body surface. Each part’s injury degree is scored on a scale of 1 to 6 points, with the six categories being mild, moderate, severe but not life-threatening, severe but life-threatening but survivable, extremely severe and unable to rescue success. The ISS had an effective range of 1–75 points. A higher score indicated a more serious injury and a lesser chance of survival [31].

#### Blood culture processing

In cases of BSI, both aerobic and anaerobic BACTEC blood culture bottles were inoculated with blood and incubated in the BD BACTEC automated system for 5 days. When the blood culture bottle flagged positive, then Gram stain and culture were performed by inoculation onto culture media such as blood agar and MacConkey agar. Organism identification was done using MALDI-TOF MS, and antibiotic susceptibility testing was performed.

#### Sepsis criteria

According to the Surviving Sepsis Campaign Bundle, blood culture indicators include fever [body temperature (BT) 101–102.9°F] with a sepsis-related sign, such as shivering; more than 5 days of indwelling central venous catheters; a white blood cell (WBC) count of >18 000 cells/mm^3^; a systolic blood pressure (SBP) of less than 90 mmHg and an infection that is not explained [32].

The SIRS criteria use the clinical criteria of surviving sepsis campaign for severe sepsis [33] with the presence of at least two of the following: heart rate >90 beats/min, respiratory rate ≥22 breaths/min, BT >38 °C or BT <36 °C, WBC >12 000 or less than 4000 cells/mm^3^.

Sepsis was defined in those patients who had a score of ≥2 in the SIRS criteria with positive microbial cultures, and severe sepsis was defined as sepsis with organ failure. We have also calculated the qSOFA score for each patient. The score ranges from 0 to 3 with one point allocated for each of the following clinical signs: SBP <100 mmHg, respiratory rate ≥22 /min and altered mental status (GCS score <15) from baseline. A score of ≥2 indicates more severity with increased ICU length of stay and mortality [7].

### Bias

All patients meeting the inclusion criteria during the study period were included in this study, thereby reducing the chances of selection bias.

### Study size

The study size was determined based on a study conducted by Mathew *et al*. (2022), and the incidence rate of wound swab bacterial growth was 64%. The sample size was estimated using software power analysis and sample size, version 16 (PASS-16).

### Quantitative variables

The patient’s file provided information on patient demographics, comorbidities, trauma characteristics, interventions and complications during hospital stays and other outcomes, such as hospital disposition and duration of stay.

### Statistical methods

Values were calculated as mean, median and interquartile range. In the analysis of risk factors for sepsis, the comparison between groups for categorical variables was estimated by using χ^2^ tests. All statistical analyses were performed using SPSS statistical software (IBM SPSS version 20; Armonk, NY), with a *P* value <0.05 considered statistically significant.

## Results

### Participants

The study included 189 RTA patients meeting the inclusion criteria. Culture-confirmed cases of wound swabs or aspirate samples were 99 (52.38%) out of 189 over the duration of the study period (aerobic organism = 97 and anaerobic organism = 02). Of the 189 RTA patients, sepsis developed in 50 (26.45%) patients. Among the sepsis patients, 30 (60%) patients have the culture-positive wound swab.

### Descriptive data

The median age of the study population was 31 years. Most of the patients belonged to the age group of 20–30 years (49.09%). Males (*n* = 158, 83.59%) suffered a greater number of RTA injuries than females. Mortality was attributed to wound infection in 22 (11.64%) patients during the hospital course.

### Main results

**Fig. 2.**
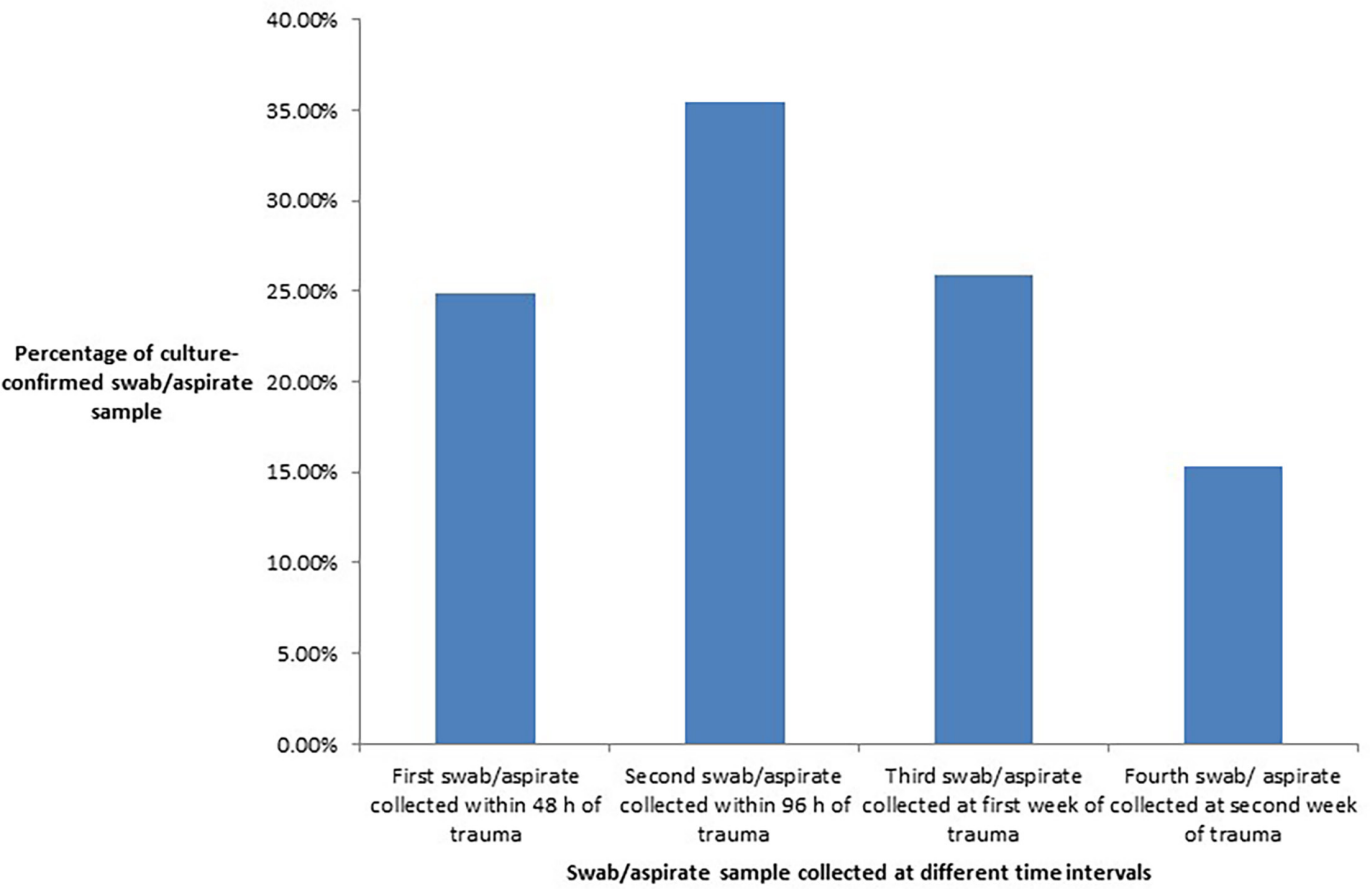
Column graph showing the percentage positivity of cultured wound deep swabs or aspirate samples collected at different time intervals.

The culture positivity of different wound deep swabs or aspirate samples collected at different time intervals from RTA patients was evaluated (Fig. 2). The most common positive wound deep swab or aspirate sample was the second sample, which was collected within 96 h of trauma, followed by the third sample, which was collected in the first week after traumatic injury. The isolated microorganisms from the wound sample constituted 28 Gram-positive cocci (28.28%), 69 (69.69%) Gram-negative bacilli and 2 (2.02%) anaerobic microorganisms (Table 1). Among the Gram-negative bacteria, the most commonly isolated microorganisms were *Klebsiella pneumoniae* (17/69, 24.63%) and *Pseudomonas aeruginosa* (15/69, 21.73%), and among the Gram-positive bacteria, *S. aureus* (19/28, 67.85%) was the predominantly isolated microorganism. Ninety-nine (52.38%) patients developed wound infection, which was defined by the culture positivity of the wound swab as well as the clinical signs and symptoms, thereby differentiating between infection and colonization. Anaerobic microorganisms were isolated from only two samples (2.02%). They were *Bacteroides fragilis* (*n* = 1) and *Clostridium perfringens* (*n* = 1).

**Table 1.** Table showing the isolated microorganisms from aerobic wound culture isolates (*n* = 97)

Isolated organisms	No.	%
*Staphylococcus aureus*	19	19.58
*Enterococcus faecalis*	5	5.15
*Enterococcus faecium*	4	4.12
*Klebsiella pneumoniae*	17	17.52
*Pseudomonas aeruginosa*	15	15.46
*Escherichia coli*	13	13.40
*Enterobacter aerogenes*	3	3.09
*Enterobacter cloacae*	6	6.18
*Klebsiella oxytoca*	3	3.09
*Proteus mirabilis*	3	3.09
*Acinetobacter baumannii*	4	4.12
*Acinetobacter lwoffii*	1	1.03
*Providencia rettgeri*	1	1.03
*Citrobacter koseri*	1	1.03
*Citrobacter freundii*	1	1.03
*Proteus vulgaris*	1	1.03

**Fig. 3.**
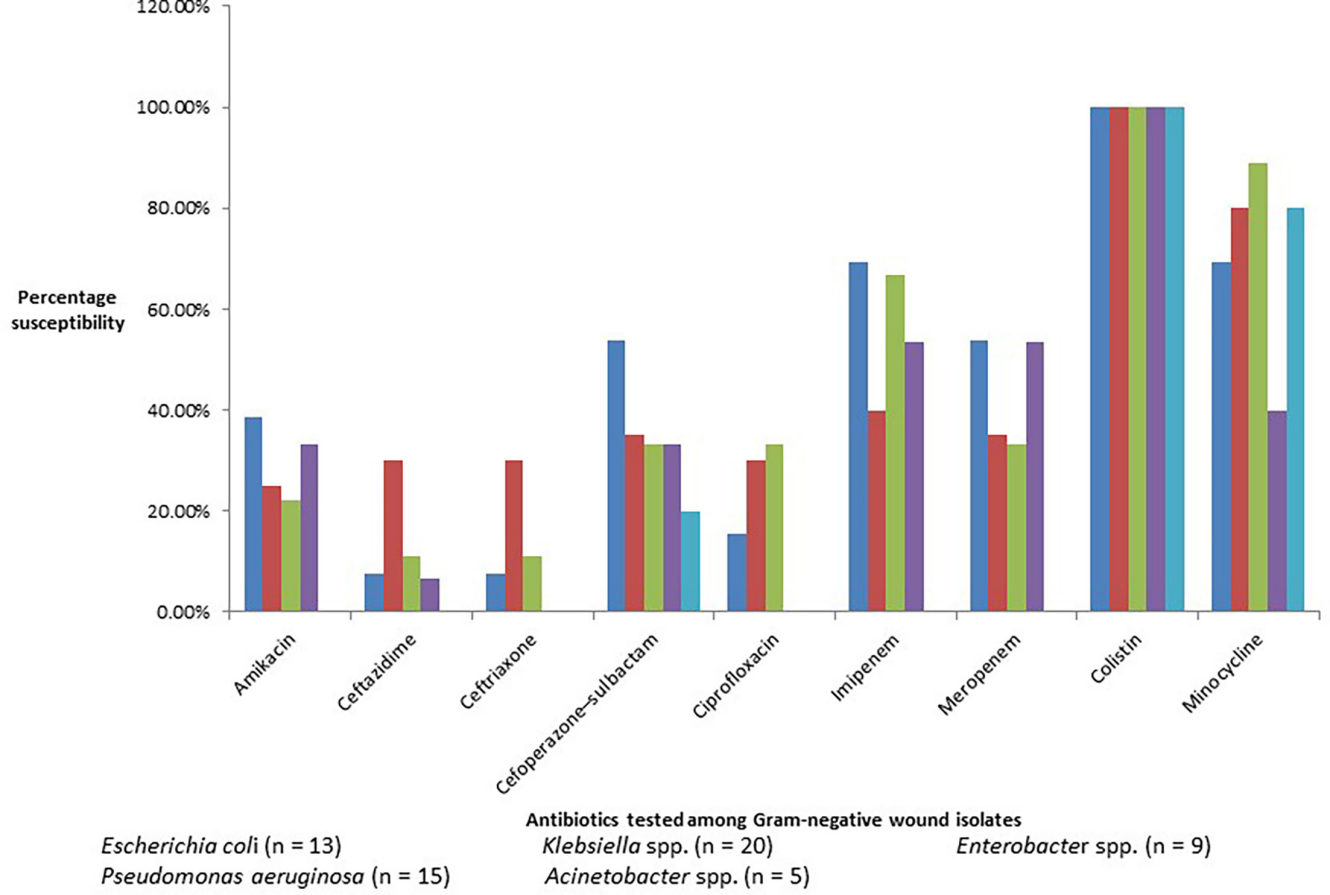
Column graph showing the susceptibility pattern to different antibiotics among the commonly isolated Gram-negative aerobic wound isolates.

Most of the Gram-negative isolates were resistant to third-generation cephalosporin and fluoroquinolones (Fig. 3). Only 30% susceptibility was seen for third-generation cephalosporin and ciprofloxacin, 25% for amikacin, 35% for cefoperazone–sulbactam and 40% for imipenem in *Klebsiella* spp. (*n* = 20) wound isolates. *P. aeruginosa* wound isolates (*n* = 15) showed only 6% susceptibility for ceftazidime, 20% for piperacillin–tazobactam and 33% for levofloxacin and amikacin. Among the *Escherichia coli* isolates (*n* = 13), only 7% susceptibility was seen for third-generation cephalosporin and 15% for ciprofloxacin. All the *Acinetobacter* spp. wound isolates were resistant to amikacin, third-generation cephalosporin, fluoroquinolones and carbapenem. None of the Gram-negative isolates were resistant to colistin.

**Fig. 4.**
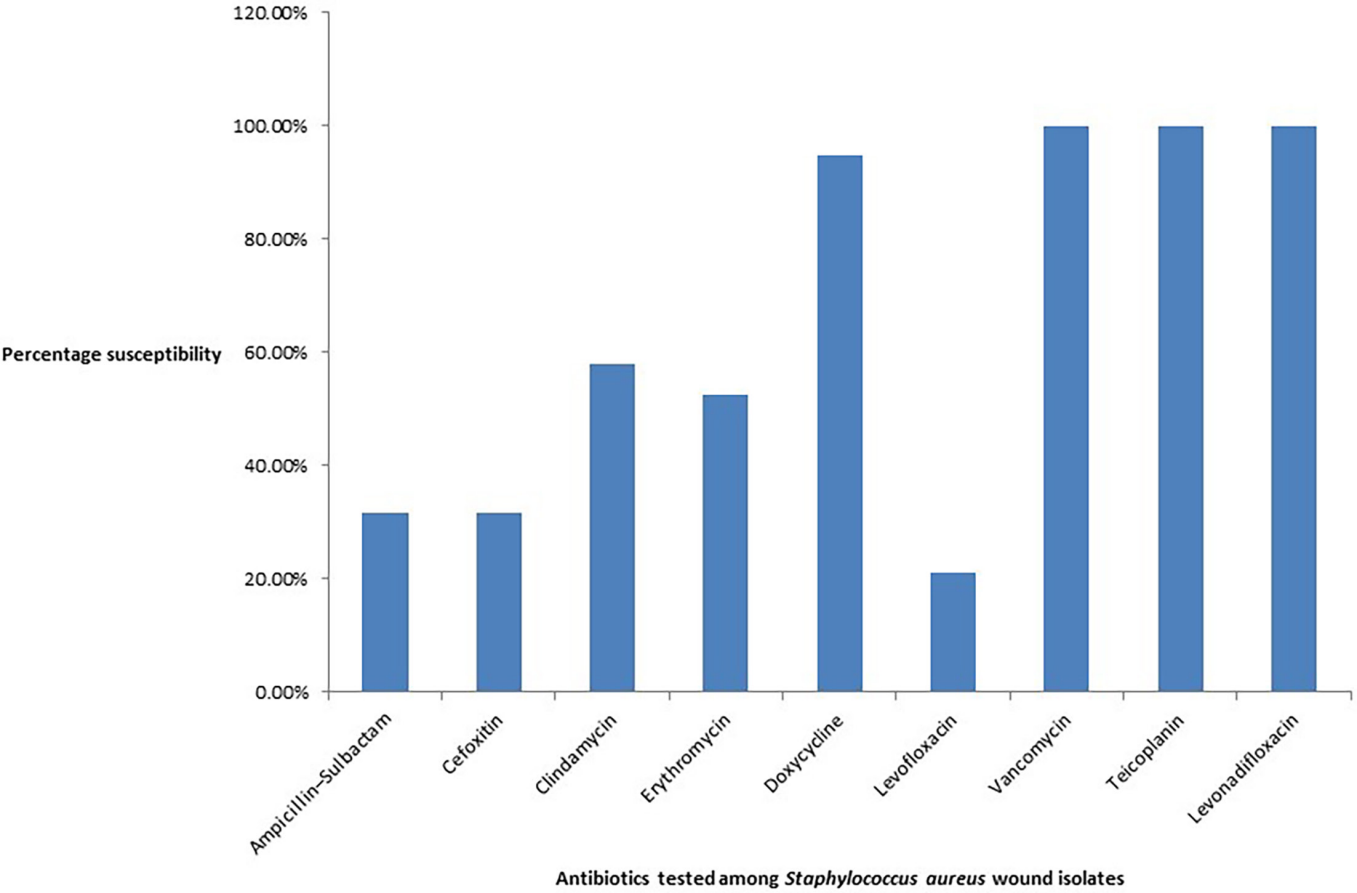
Column graph showing the percentage susceptibility to different antibiotics among *Staphylococcus aureus* wound isolates (*n* = 19).

*S. aureus* isolates were mostly resistant to cefoxitin, ampicillin–sulbactam and levofloxacin. All the *S. aureus* isolates were susceptible to vancomycin, teicoplanin and levonadifloxacin. Fig. 4 shows the antibiotic susceptibility pattern among the *S. aureus* wound isolates. *Enterococcus faecalis* (*n* = 5) wound isolates showed only 20 % susceptibility for ampicillin, ampicillin–sulbactam, doxycycline and levofloxacin. All the *Enterococcus faecium* (*n* = 4) wound isolates were resistant to ampicillin, ampicillin–sulbactam, levofloxacin and high-level gentamicin.

Carbapenem resistance among Gram-negative isolates was 52.17% (36/69), of which 25 were Enterobacterales, 5 were *Acinetobacter* spp. and 6 were *P. aeruginosa*. Also, 68.42% of the *S. aureus* isolates were found to be methicillin-resistant. Among the four isolated *E. faecium* species, the three isolates were resistant to vancomycin (Vancomycin resistant Enterococci, VRE). MDRO among the isolates was 61 (62.88%), of which 45 were Gram-negative isolates and 16 were Gram-positive isolates, suggesting increasing antibiotic resistance.

**Fig. 5.**
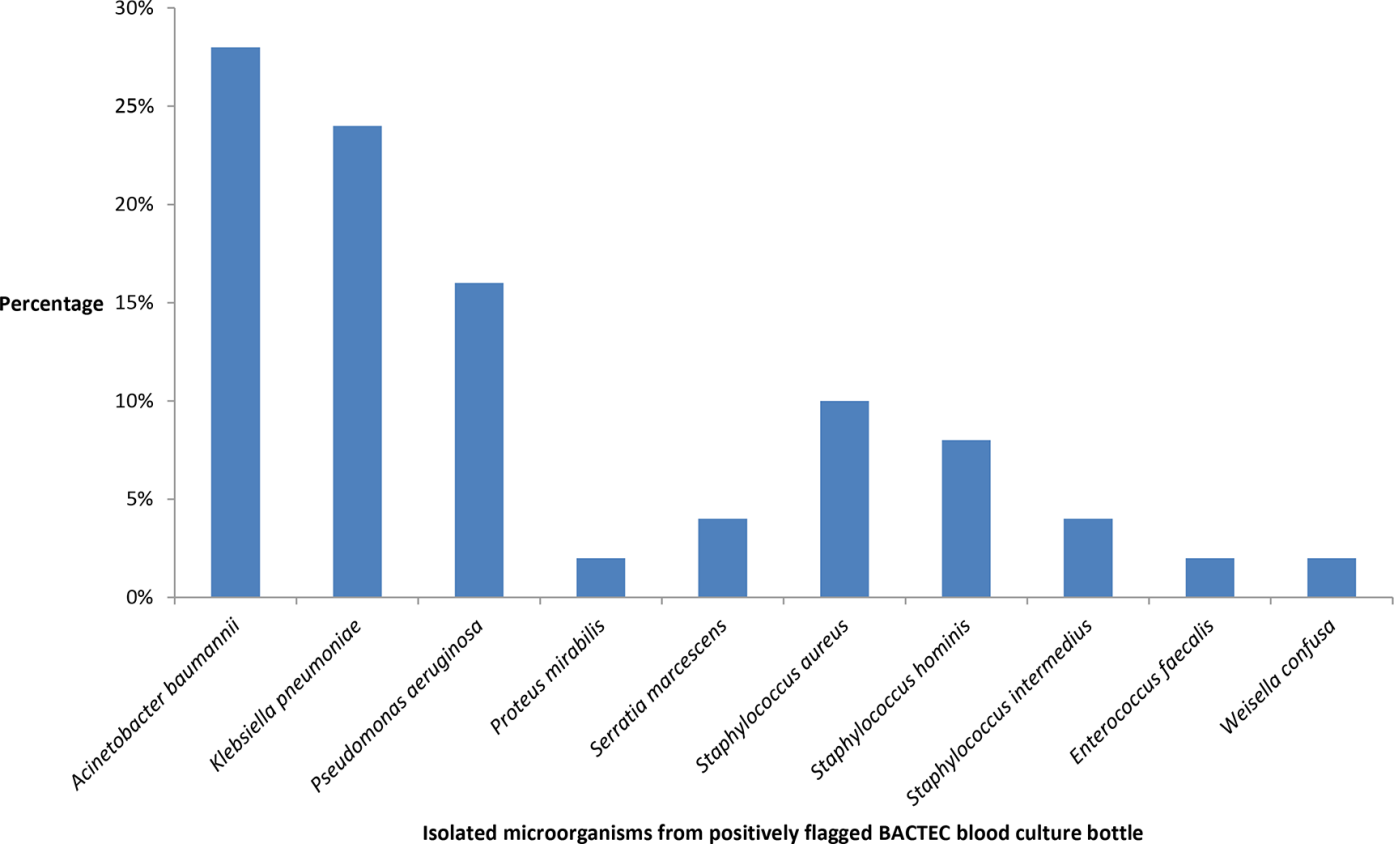
Column graph showing the isolated microorganisms from positively flagged BACTEC blood culture bottles (*n* = 50).

Fig. 5 shows the isolated microorganisms from positively flagged BACTEC blood culture bottles. *Acinetobacter baumannii* (14/50, 28%) followed by *K. pneumoniae* (12/50, 24%) were the most common isolated microorganisms in sepsis patients. Higher mortality was seen among sepsis patients, *n* = 11 (22 %) compared to non-sepsis patients, *n* = 11 (7.19%). In patients with * A. baumannii* in BSI, only 4 patients’ wound isolates showed the growth of *A. baumannii*, and in the rest of the 10 patients, the focus of infection was the respiratory tract and abdominal cavity.

Sepsis developed in 50 RTA patients during the study period, and its diagnosis was established in those patients who had ≥2 score in the SIRS criteria along with a positive microbial culture. The demographic profile and clinical characteristics of RTA patients were also evaluated (Table 2). In this study, we found that the sepsis group of patients had a higher ISS, a low GCS score, low blood pressure, higher mortality, a higher qSOFA score, an increased blood transfusion requirement and a longer length of hospital stay.

**Table 2.** Table showing the demographics and clinical characteristics of RTA patients (*n* = 189)

Variables	Sepsis(*n* = 50)	No sepsis(*n* = 139)	***P*-value**
Age ≥60 years	2	11	0.3577
Male sex	42 (84%)	116 (83.45%)	0.9287
SBP <100 mmHg	19	41	0.2693
Diastolic blood pressure <60 mmHg	12	25	0.3596
Pulse rate ≥90 (/min)	24	40	0.0149
Respiratory rate >22 (/min)	19	33	0.0550
ISS ≥9	34	21	<0.0001
GCS <8	36	23	<0.0001
RBC transfusion requirement	31	91	0.6604
**Wound**			
Abrasion	21	57	0.9027
Laceration	18	48	0.8519
Open wound	11	34	0.7262
qSOFA score	31	29	<0.0001
Mortality	11 (22%)	11 (7.19%)	0.0104
LOS >30 days	17	21	0.0053

LOS, length of stayRBC, red blood cell

Table 3 shows the various risk factors found to be statistically significant between the sepsis (*n* = 50) and non-sepsis (*n* = 139) groups of RTA patients. Moreover, 62 % of sepsis patients have qSOFA scores ≥2 compared to 21 % among the non-sepsis group of patients. PCT levels were raised in 64% of sepsis patients and in 26% of non-sepsis patients. The ISS was higher in 68% of sepsis patients and 16% of non-sepsis patients. The GCS score was low in 72 % of sepsis patients and in 16 % of non-sepsis patients. Also, 48% of sepsis patients and 27% of non-sepsis patients required ventilation. Therefore, the risk factors evaluated as significant in this study were a higher qSOFA score, a high PCT level, a higher ISS, a low GCS score and the need for mechanical ventilation. Blood transfusion was required by both the sepsis and non-sepsis groups of patients, but in sepsis patients, more units of blood were transfused. Male sex was equally affected in the sepsis and non-sepsis groups of RTA patients, so blood transfusion and male sex were not found to be statistically significant in this study.

**Table 3.** Table showing the risk factors evaluated among the sepsis and non-sepsis groups of RTA patients as significant (*P* value <0.05)

Evaluated risk factor	Sepsis (*n* = 50) patients	Non-sepsis (*n* = 139) patients	***P*-value**
PCT level ≥2 µg/l	32	37	<0.0001
ISS ≥9	34	21	<0.0001
GCS <8	36	23	<0.0001
qSOFA score ≥2	31	29	<0.0001
Need for mechanical ventilation	24	38	0.0085

**Fig. 6.**
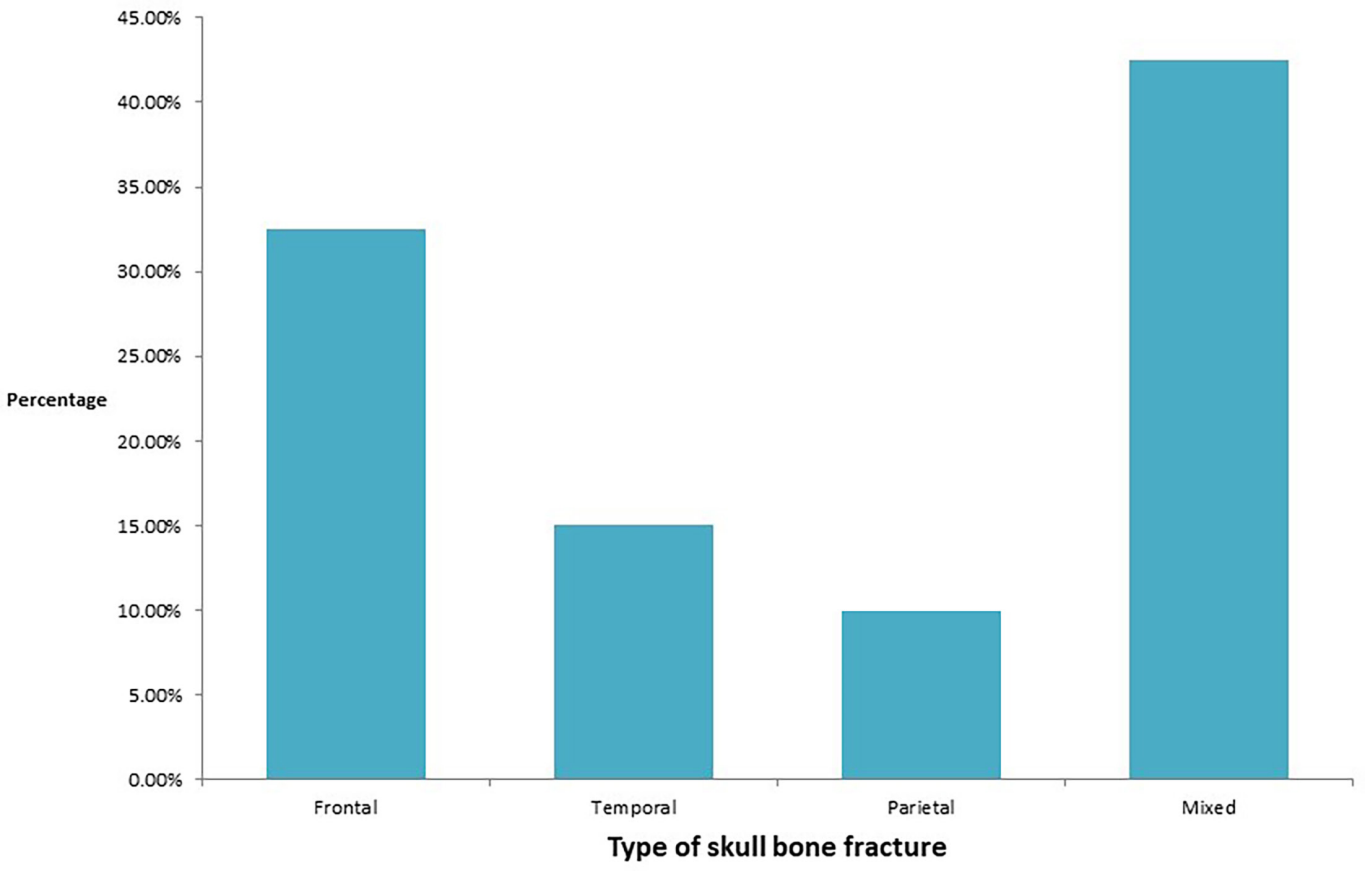
Column graph showing the various types of skull bone fractures (*n* = 120).

The pattern of skull bone fracture was also evaluated (Fig. 6), as most of the patients presented with head injuries (*n* = 120, 63.49%). The most common skull bone fracture was mixed mainly fronto-temporal, followed by a frontal bone fracture. Various comorbidities were present in RTA patients, of which the most common were diabetes mellitus (33/189, 17.46%) and hypertension (22/189, 11.64%), followed by acute kidney injury (4/189, 2.11%). During hospitalization, leucocytosis with neutrophilia was observed in 133 (70.37%) patients. Seventy-eight (41.26%) patients have developed anaemia and 83 (43.91 %) patients have developed thrombocytopaenia. Ninety-four (49.73%) RTA patients required blood transfusions.

## Discussion

In addition to the type and extent of the injury, the outcome of trauma patients was determined by the standard of acute care and the incidence of complications. In the current study, the most commonly affected RTA patients belonged to the age group of 20–30 years. This finding was similar to the study where 49% of patients were in the 16–30 year age group [34]. In addition, 83.59 % of our study population were males, who were more commonly affected by RTA injuries due to more outdoor activities, and this was similar to other studies carried out by Wong *et al.* and Jha *et al.* [35,36]. The most common types of head injury seen in this study were abrasion and laceration, which were similar to the study by Jha *et al.* [36]. All patients meeting the inclusion criteria were included in this study, thereby reducing the chances of selection bias and ensuring that the sample obtained was representative of the population.

In our study, 52.38% of patients had grown a pathogen among wound isolates. A study conducted by Mathew *et al.*, showed that 64% was the infection rate of wound swabs [19]. In our study, aerobic wound sample isolates constituted 97.97 % and anaerobic isolates constituted 2.02 %. The study conducted by Brook and Frazier isolated the growth of 444 anaerobic and 267 aerobic isolates, which differed from our study [20].

In the present study, the most common positive wound deep swab or aspirate sample was the second sample, followed by the third sample, suggesting nosocomial infection among hospitalized patients. To the best of our knowledge, this was the first study that has done a comparative analysis of the positivity of different wound samples collected at different time intervals among RTA patients. With the follow-up of the subsequent sample to study the change in the bacterial aetiology, we have found that only two patient wound swabs yielded two different organisms, while the rest of the swabs or aspirates yielded the same organism with the same antibiogram in the subsequent sample. In one patient, the first and second swab cultures showed the growth of *S. aureus*, but the third swab culture showed the growth of *K. pneumoniae*. In another patient, second and third swab cultures showed the growth of *K. pneumoniae* and a fourth swab yielded *A. baumannii*.

This study showed that the most commonly isolated microorganism from the wound sample was the Gram-negative bacilli, among which *K. pneumoniae* and *P. aeruginosa*, followed by *E. coli*, were the predominant microorganisms, and this was similar to the study conducted by Mathew *et al.* [19]. Among the Gram-positive microorganisms, *S. aureus* was the predominant microorganism causing wound site infection, which was similar to the study conducted by Mythri *et al.* [37].

This study has demonstrated that patients treated with injuries frequently have MDRO, and these infections were linked to a poor prognosis. The identification of bacteria resistant to carbapenem (52.17%) among the Gram-negative bacteria and more than three classes of regularly used medicines (62.88%) among the Gram-positive and Gram-negative bacteria was a concerning finding of this investigation, suggesting increasing antibiotic resistance. Every critical care physician in the world is concerned about drug resistance, especially in light of the possibility of very modest increases in the armament of antibiotics [38,40].

In our study, *Klebsiella* spp. wound isolates showed only 25% susceptibility for amikacin and 30% for third-generation cephalosporin and ciprofloxacin, whereas for *P. aeruginosa*, only 33% susceptibility was seen for amikacin and 7% for ceftazidime. None of the *Acinetobacter* spp. isolates were susceptible to amikacin, third-generation cephalosporin, ciprofloxacin and carbapenem. A study by Mathew *et al.* showed that 65 and 29 % of *Citrobacter* species isolates were sensitive to amikacin and ciprofloxacin, respectively, whereas 29% of *E. coli* was sensitive to cefuroxime, amikacin and ciprofloxacin [19]. Similarly, percentages of *Acinetobacter* species were sensitive to ceftriaxone and amikacin [19].

*S. aureus* isolates showed 21% susceptibility to levofloxacin and 32% susceptibility to cefoxitin and ampicillin–sulbactam. All the *E. faecium* isolates were resistant to ampicillin, levofloxacin and high-level gentamicin. In another study, *Enterococcus* species were found to be more sensitive towards amikacin (40%) and *S. aureus* was found to be more sensitive to cefuroxime (44%) and amikacin (22%) [19].

A low GCS, a raised PCT level, a higher ISS, the need for mechanical ventilation and a raised qSOFA score were associated factors for sepsis in the current study. As observed in the current study, sepsis developed in 26.45% of the study patients, and the mortality rate was 22% (*n* = 11) among the sepsis patients, which was different from the study conducted by Park *et al.*, showing that the incidence of sepsis was 8.2% and mortality was 6.7% in trauma patients [40]. Our results are in line with the study conducted by Shankar-Hari *et al.*, showing 22.4% mortality in ICU patients with sepsis [23].

Other studies showed that sepsis might be predicted independently by ISS, RTS, admission GCS, concomitant conditions (diabetes, heart disease and immunological disorder, for example), male sex, RBC transfusion and age [24,41]. In our study, male sex and blood transfusion were not found to be statistically significant, which was different from the previous study performed in sepsis patients.

In our study, qSOFA and SIRS criteria were the important predictors for the diagnosis of sepsis, along with a positive culture. Our results are in line with other studies, which show that qSOFA predicts critical illness as well as or better than SIRS [42]. These findings were dissimilar to those of other studies. [43,44]

The most commonly isolated microorganisms from the BSI were the Gram-negative bacilli, which was similar to the study performed by Jain *et al.* and Agrawal *et al.* [21,22].

Our study has some limitations. First, we isolated only two anaerobic microorganisms, which were due to the lack of the optimum anaerobic facility. Second, it was a single-centre study with few isolates that do not represent the picture of infections in the other trauma care centres in India.

## Conclusion

It is important to ascertain the antibiogram of the isolated microorganisms as soon as possible in order to prevent unnecessary extended empirical therapy and to deliver suitable and efficient treatment. For trauma patients, sepsis continues to be a major concern. Therefore, risk factors for sepsis should be evaluated, which will allow clinicians to be more vigilant in identifying patients who may deteriorate and allow more prompt intervention. SIRS criteria and qSOFA score are better predictors for the early diagnosis of sepsis with a positive microbial culture. The most commonly isolated microorganisms from the wound sample were *K. pneumoniae* and *P. aeruginosa* followed by *E. coli* among the Gram-negative bacteria and *S. aureus* among the Gram-positive bacteria. None of the Gram-negative isolates were resistant to colistin, and all the Gram-positive isolates were susceptible to vancomycin, teicoplanin and levonadifloxacin. The most resistant bacteria among the Gram-negative isolates were *Acinetobacter* spp., which was resistant to all the first-line antibiotics. Gram-negative bacteria were mostly resistant to third-generation cephalosporin and fluoroquinolones. Most of the *Enterococcus* spp. were resistant to ampicillin, levofloxacin, ampicillin–sulbactam and doxycycline.

## Abbreviations

ATC: Apex Trauma Centre
BSI: bloodstream infection
BT: body temperature
CLSI: Clinical Laboratory Standards Institute
GCS: Glasgow Coma Scale
ICU: intensive care unit
IEC: Institutional Ethics Committee
ISS: injury severity score
LOS: length of stay
MDROs: multidrug-resistant organisms
MOF: multiple organ failure
PCT: procalcitonin
qSOFA: quick sequential organ failure assessment
RBC transfusion: red blood cell transfusion
RTA: road traffic accident
RTS: revised trauma score
SBP: systolic blood pressure
SIRS: systemic inflammatory response syndrome
WBC: white blood cell
